# Editorial: Plant RNA Biology

**DOI:** 10.3389/fpls.2019.00887

**Published:** 2019-07-09

**Authors:** Dóra Szakonyi, Ana Confraria, Concetta Valerio, Paula Duque, Dorothee Staiger

**Affiliations:** ^1^Instituto Gulbenkian de Ciência, Oeiras, Portugal; ^2^RNA Biology and Molecular Physiology, Faculty of Biology, Bielefeld University, Bielefeld, Germany

**Keywords:** posttranscriptional regulation, RNA processing, RNA stability, non-coding RNAs, RNA structure, transcriptomics

Initially regarded as mere intermediates between coding DNA sequences and proteins, RNAs have been ascribed a plethora of novel roles in the past decades. Today, they are recognized as active regulatory molecules, influencing processes such as gene expression, chromatin organization or genome stability that affect all aspects of a plant's life. This Research Topic aims at highlighting the broad biological significance of RNAs in plant systems and contains eight original research articles, one review and four mini-reviews, covering various RNA-based mechanisms in higher plants.

As they are being synthesized, RNAs fold into secondary structures, which can influence RNA metabolism at multiple levels, including transcript processing and stability, or protein translation. Yang et al. not only bring attention to the significance of RNA structures, but also summarize the currently available methods to study the folding of specific RNA molecules or profile genome-scale RNA structuromes.

After transcription, RNAs require proper maturation through a multi-step process integrating capping, splicing, and polyadenylation. Pieczynski et al. describe the importance of accurate splicing of the precursor mRNA (pre-mRNA) encoding the small subunit of the Arabidopsis nuclear cap-binding complex, *CBP20*, which contains seven introns. Of these, intron 4 belongs to the U12 class of introns and separates the gene into an N-terminal half encoding the RNA-binding moiety and a C-terminal half encoding a nuclear localization signal. Using intron swapping and mutagenesis, the authors demonstrate that the U12 intron is crucial for correct *CBP20* splicing. Mitochondrial and chloroplastidial transcripts also undergo processing, which in the chloroplast is largely carried out by imported nuclear-encoded RNA-binding proteins and RNA-binding factors encoded by the organellar genome. Chloroplasts of land plants retain a single plastid-encoded splicing factor, intron maturase MatK. Qu et al. ectopically expressed MatK in tobacco chloroplasts and observed variegation of the cotyledons, indicating that adequate MatK levels are required for proper chloroplast development. Interestingly, the splicing pattern of selected plastid genes was not strongly affected in these lines, but rather translation was inhibited, leading to reprogramming of chloroplast gene expression.

Alternative splicing, which results from differential splicing of the same pre-mRNA, represents a major source of transcriptome and proteome diversity and an important regulation mechanism for development and stress responses in plants. Szakonyi and Duque scrutinize the role of alternative splicing during early plant development. They review the current knowledge on how alternative splicing changes during seed maturation, germination, and seedling formation in the dark and light. Furthermore, they compile the splicing factors with crucial roles during these initial steps of development and the alternatively-spliced genes with putative functional roles for different splice forms.

Alternative splicing or mutations can introduce premature termination codons (PTCs) that target mRNAs to degradation by an evolutionarily-conserved process termed nonsense-mediated decay (NMD). The work presented by Capitao et al. focuses on SMG7 proteins, conserved components of the NMD machinery in eukaryotes. Plants lost several core NMD genes during evolution, but *SMG7* genes expanded and acquired novel functions. In contrast to previous findings, the authors report that *SMG7* is not an essential gene in Arabidopsis and fulfills an additional, NMD-unrelated function in meiosis. Moreover, *SMG7-like* (*SMG7L*), a close paralog arising from gene duplication in dicots, is not functionally redundant with *SMG7* though its biological role remains unclear.

Research in the last couple of decades has also uncovered several different types of non-coding RNAs, grouped according to size into long non-coding (lnc) RNAs and a highly diverse pool of small RNAs. The latter can be further classified depending on their biogenesis pathway and include microRNAs (miRNAs), encoded by *MIR* genes, as well as several classes of short interfering RNAs (siRNAs), generated from double-stranded RNA (Borges and Martienssen, 2015). Non-coding RNAs exert different regulatory functions in plants, playing important roles in developmental programs but also integrating environmental cues and participating in stress responses (Chekanova, 2015; D'ario et al., 2017; Li et al., 2017; Sun et al., 2018). In this context, Sánchez-Retuerta et al. summarize the current knowledge on light regulation of miRNAs and lncRNAs and their functions in photomorphogenesis, circadian clock regulation, and photoperiod-dependent flowering. On the other hand, the review by Cho highlights the potential role of transposon-derived lncRNAs and small RNAs, which suppress harmful effects of transposition, but can also act on non-transposon transcripts affecting development and stress responses. Furthermore, Calixto et al. analyze the effect of cold stress on the expression and processing of regulatory non-coding RNAs, performing an ultra-deep sequencing of a diel time-series of Arabidopsis plants. The authors identified specific lncRNAs and primary miRNAs differentially expressed and/or alternatively spliced in response to cold stress that potentially contribute to acclimation and freezing tolerance. Bazin et al. explore the role of Nuclear Speckle RNA-binding proteins (NSRs), which are known alternative splicing modulators, through a regulatory loop involving the lncRNA *Alternative Splicing Competitor RNA (ASCO)* (Bardou et al., 2014). Using RNA immunoprecipitation coupled with RNA-seq, the authors showed that NSRs directly interact with lncRNAs, many of which are upregulated in the *nsra nsrb* mutant. This suggests that NSRs regulate the steady-state abundance of lncRNAs, which in turn may contribute to splicing regulation.

In the plant cell nucleus, miRNAs are processed from primary miRNA transcripts by the Microprocessor complex, which is composed of three core proteins in Arabidopsis: Dicer-Like 1 (DCL1), Hyponastic Leaves 1 (HYL1), and Serrate (SE) (Song et al., 2019). Dolata et al. review how the Microprocessor complex is regulated, emphasizing the posttranslational regulation of its components, but also covering posttranscriptional regulatory mechanisms. Chitarra et al. reveal miRNA changes associated to development, photosynthesis, jasmonate signaling, and disease resistance in *Vitis vinifera* infected with the phytoplasma *Flavescence dorée*. Importantly, the authors provide a valuable tool for the research community working on *Vitis* spp. (miRVIT, available at http://mirvit.ipsp.cnr.it/); they assemble a comprehensive database of novel putative gravepine miRNAs, uniformly reannotating and aligning all described accessions to the latest version of the grape genome and listing their validated and predicted target transcripts. In common bean, Martin-Rodriguez et al. explore the role of the nodule-expressed miR319d in the regulation of rhizobial infection and symbiotic nodule development. The authors identified the transcription factor *TEOSINTE BRANCHED/CYCLOIDEA/PCF 10* (*TCP10*) as a target of miR319d and propose that the effect of miR319d-TCP10 on nodulation involves the modulation of jasmonate signaling through the transcriptional regulation of the jasmonate biosynthetic gene *LIPOXYGENASE 2* (*LOX2*). The 5′ fragments of ARGONAUTE 1 (AGO1)-mediated cleavage guided by miRNAs and siRNAs are uridylated at the 3′ end to facilitate further degradation. In Arabidopsis, uridylation of these fragments is carried out by two terminal uridylyltransferases (TUTases) HEN1 SUPPRESSOR 1 (HESO1) and URIDYLYLTRANSFERASE 1 (URT1), whose activity is explored in the study of Zuber et al. The authors optimize a 3′RACE-seq method to analyze quantitatively and qualitatively the uridylation at the 3′ ends of AGO1 5′ cleavage products. Showcasing the applicability of 3′RACE-seq, the 5′ cleavage fragments of the miR159 target *MYB33* and the miR156/157 target *SQUAMOSA-PROMOTER BINDING PROTEIN-LIKE 13* (*SPL13*) are compared in the wild type and mutants impaired in TUTase activity. The authors uncover different contributions of HESO1 and URT1 to the uridylation of the cleavage fragments, with HESO1 being the main TUTase.

The diverse collection of articles in this Research Topic demonstrates the current vibrant and rapid progression of the plant RNA biology field. Current major challenges include, but are not limited to, identifying functional protein-RNA interactions (Foley et al., 2017), ascribing biological functions to different RNAs (Zhao et al., 2019), and dissecting tissue complexity using single cell transcriptomics (Ryu et al., 2019). Technological advances coupled to the increase in publicly-available data are expected to allow the community to address these and other issues at a fast pace in upcoming years, bringing key novel insights into our understanding of the mechanisms through which RNA is regulated and regulates biological processes in plant cells.

## Author Contributions

All authors listed have made a substantial, direct and intellectual contribution to the work, and approved it for publication.

### Conflict of Interest Statement

The authors declare that the research was conducted in the absence of any commercial or financial relationships that could be construed as a potential conflict of interest.
